# Optimization of Bacterial-to-Cementation Solution Ratio for MICP-Treated Sand: Effects on Compressibility and Slope Erosion Resistance

**DOI:** 10.3390/ma19132860

**Published:** 2026-07-04

**Authors:** Yanhong Li, Qian Zhang, Yunfei Huang, Yuxiang Zhang, Liquan Xie

**Affiliations:** 1 School of Ocean and Civil Engineering, Shanghai Jiao Tong University, Shanghai 200240, China; 2 Guangxi Pinglu Canal Construction Co., Ltd., Qinzhou 535000, China; 3 Guangdong Basic Engineering Group Co., Ltd., Guangzhou 510620, China; huangyunfei15@163.com; 4 College of Civil Engineering, Tongji University, Shanghai 200092, China; 2530754@tongji.edu.cn (Y.Z.); xie_liquan@tongji.edu.cn (L.X.)

**Keywords:** microbially induced calcium carbonate precipitation (MICP), sand solidification, ratio optimization, compression index, slope erosion resistance

## Abstract

**Highlights:**

- Optimal bacterial-to-cementation ratio (3:2) yields lowest compression index (0.044).
- Too high or low ratio causes sparse or agglomerated CaCO_3_, raising *C_c_* ≥ 0.064.
- Optimal ratio reduces erosion modulus by 55.0–57.5% vs. untreated slope.
- Safety factor K increases from 3.97 to 5.19 as solidification improves.
- Soil pH after treatment (5.59–6.32) is within vegetation tolerance range.

**Abstract:**

In engineering applications such as filling and slope protection, natural river sand suffers from high compressibility and poor erosion resistance. Microbially induced calcium carbonate precipitation (MICP) can mitigate these issues by sand solidification, but the optimal volumetric ratio of bacterial solution to cementation solution ($r_{v}$) for natural river sand remains unclear. This study used natural river sand (0.063–1.6 mm), Bacillus subtilis, and a cementation solution (2 M urea + 2 M $Ca{Cl}_{2}$, 1:1). Eight $r_{v}$ values from 2:5 to 3:1 were tested. Compressibility was evaluated via one-dimensional consolidation tests, and erosion resistance via a slope model. Results show a non-linear “U-shaped” relationship between $r_{v}$ and compression index ($C_{c}$). The optimal $r_{v}$ = 3:2 yields the lowest $C_{c}$ (0.044). Higher or lower ratios increase $C_{c}$ to ≥0.064. Microscopy reveals that at $r_{v}$ = 3:2, a dense, continuous $Ca{CO}_{3}$ network fills pores, whereas excess bacteria cause sparse cementation and too few cause local agglomeration. The optimal ratio reduces erosion modulus by 55.0–57.5% compared to untreated slopes. This work provides a quantitative, eco-friendly optimization strategy for MICP-treated natural river sand, balancing mechanical performance with ecological adaptability (pH within vegetation tolerance).

## 1. Introduction

Natural river sand is widely used in geotechnical engineering for embankment fills, foundation layers, and slope protection. However, its loose granular structure typically results in high compressibility and poor resistance to hydraulic erosion, leading to uneven settlement and slope instability [1,2,3]. Traditional improvement methods such as cement grouting or mechanical compaction are energy-intensive and may disturb the environment. In this context, microbially induced calcium carbonate precipitation (MICP) has emerged as a sustainable bio-mediated alternative [4,5]. MICP uses urease-producing bacteria to hydrolyze urea, generating carbonate ions that precipitate calcium carbonate (CaCO_3_) within the soil matrix, thereby binding sand particles and enhancing mechanical properties [6,7].

A large body of research has demonstrated the potential of MICP for ground improvement and erosion mitigation. Shukla and Sharma reviewed recent developments in using microorganisms for geotechnical applications, highlighting the eco-friendly nature of MICP [8]. Jiang et al. provided a comprehensive introspection into MICP processes, materials, characterization, and applications [9]. Harran et al. discussed breakthroughs and challenges in the mechanics, modeling, and upscaling of biocemented soils [10]. Payan et al. specifically reviewed the application of MICP for soil erosion mitigation as a sustainable approach [11]. For riverbank and slope protection, Salifu et al. showed that MICP can stabilise sandy foreshore slopes [12], while Yang et al. demonstrated its effectiveness in mitigating hydraulic erosion of riverbanks [13]. Li et al. reported improved deformation characteristics and stability of ecological geobag revetments treated with MICP [14]. Clara Saracho et al. used flume tests to study the effects of MICP on the erosional behaviour of fine sand [15]. Moreover, Cui et al. found that MICP reduces sandbank dust emission during flood recession [16], and Jiang et al. confirmed the applicability of microbial calcification for sandy-slope surface erosion control [17].

In addition to erosion resistance, the mechanical behaviour of MICP-treated sands, especially compressibility, is critical for engineering fills. Xiao et al. investigated the compression behaviour of MICP-treated sand with various gradations, noting that uniform CaCO_3_ distribution is key [18]. Cui et al. examined the influence of cementation level on the strength behaviour of bio-cemented sand [19]. Zeitouny et al. studied the impact of a new combined treatment method on mechanical properties and microstructure [20]. For sand-silt mixtures, Rawat and Satyam assessed the effectiveness of MICP under varying silt content, cementation solution, and treatment cycles [21]. Khaleghi et al. compared Sporosarcina urea and Bacillus subtilis for dune sand bio-improvement, showing that Bacillus subtilis produces effective CaCO_3_ precipitation [22]. Reed explored the role of granular characteristics on MICP-treated sands, emphasising that precipitation patterns depend heavily on treatment conditions [23]. Kang et al. studied uniformity of MICP-solidified fine-grained silt, and found that non-uniform cementation often results from suboptimal solution ratios [24]. Furthermore, Alam and Motamed highlighted the role of MICP treatment area in mitigating liquefaction-induced settlements [25], and Zhou et al. showed through shaking table tests that MICP-treated coral sand piles improve foundation performance under seismic loading [26].

The optimisation of treatment parameters is essential for achieving desired mechanical performance while maintaining ecological compatibility. Among these parameters, the volumetric ratio of bacterial solution to cementation solution ($r_{v}$) directly influences the distribution, morphology, and total amount of CaCO_3_ precipitation. An inappropriate ratio may cause either sparse cementation (insufficient binding) or local agglomeration (clogging), both of which compromise compressibility and erosion resistance. Several recent studies have addressed this issue from different angles. He and Chu investigated undrained responses of microbially desaturated sand, noting the importance of controlled precipitation [27]. Wu et al. quantified permeability reduction in biogrounded rock fractures, indirectly highlighting the role of solution ratios [28]. Zhang et al. used DEM to provide insights into the residual strength of bio-cemented sands, showing that particle-scale cementation patterns are ratio-dependent [29]. Maroof et al. studied internal erosion on coarse alluvial sediments [30], and Jiang et al. applied MICP for seepage-induced internal erosion control in sand-clay mixtures [31], both underscoring the need for uniform cementation. Mengistu and Hordofa isolated ureolytic bacteria from cement-contaminated soil, providing insights into bacterial activity that can inform ratio selection [32]. Fukue et al. proposed a unified evaluation using optical density and carbonate precipitation rate, which can help optimise the bacterial-to-cementation ratio [33]. For expansive soil, Song et al. examined the mechanical properties and microscopic mechanism of MICP modification, revealing that CaCO_3_ morphology is highly sensitive to the treatment ratio [34]. Hussain et al. reviewed MICP for bio-concrete [35], and Joshi et al. discussed alternative phosphate precipitation methods [36], both indicating that optimisation of reagent ratios is a cross-cutting theme. Firoozi et al. emphasised the need for sustainable, optimised bio-treatment protocols to balance mechanical performance with ecological compatibility [37].

Despite these advances, a quantitative guideline for the optimal $r_{v}$ value specifically for natural river sand (0.063–1.6 mm) treated with Bacillus subtilis and a standard cementation solution (2 M urea + 2 M CaCl_2_, 1:1) is still lacking. Most previous studies focused either on compressibility or on erosion resistance separately, without a combined evaluation under the same treatment framework. Moreover, although the soil pH after treatment is important for vegetation in environmentally sensitive areas, this ecological aspect has rarely been considered alongside mechanical optimization [38,39]. This knowledge gap hinders the practical application of MICP in river training works and slope protection projects where both settlement control and erosion mitigation are required, and where ecological compatibility is essential.

Therefore, this study aims to investigate the effects of $r_{v}$ on the compressibility and slope erosion resistance of natural river sand treated with MICP. Natural river sand is solidified with Bacillus subtilis (Type II) and a urea-CaCl_2_ cementation solution. Eight $r_{v}$ values ranging from 2:5 to 3:1 are tested. Through one-dimensional solidification tests, physical slope erosion model tests, and microstructural observations, the study systematically investigates the impact of $r_{v}$ value on the compressibility index and erosion resistance of the solidified sand. The optimal ratio and quantitative parameters obtained in this work are expected to guide the application of MICP technology in natural river sand reinforcement projects.

## 2. Materials and Methods

### 2.1. Materials

Natural river sand used in this study was collected from an inland river channel. Its main component was silicon dioxide (SiO_2_). The particle size ranged from 0.063 mm to 1.6 mm. The sand was washed with tap water, dried, and stored for later use. The porosity of the sand was measured by the volume method and found to be approximately 25%. Bacillus subtilis solid-state type II was selected as the urease-producing microbial source. The viable bacterial count in the solid powder was ≥2.0 × 10^6^ CFU/g. In each experiment, the solid powder was dissolved in sterile water to prepare a bacterial suspension with a concentration of 10 g/L. The viable count of the bacterial suspension was determined by the dilution plate counting method and found to be 2.0 × 10^7^/L. The cementation solution was prepared by mixing two stock solutions of urea and calcium chloride at equal volume. First, urea (CH_4_N_2_O) and calcium chloride (CaCl_2_) were separately dissolved in deionized water to obtain 2 mol/L stock solutions. The two stock solutions were then mixed at a 1:1 volume ratio to produce a cementation solution with final concentrations of 1 mol/L urea and 1 mol/L calcium chloride. All chemicals were of analytical grade.

### 2.2. Ratio Design

To investigate the effect of $r_{v}$ on MICP solidification performance, a total of eight $r_{v}$ were designed: $r_{v}$ = 2:5, 1:2, 2:3, 1:1, 3:2, 2:1, 5:2, and 3:1. Detailed parameters for each ratio are presented in Table 1. The total volume of injected liquid in all treatment groups was fixed at one pore volume of the sand specimen. The one-pore volume was calculated from the specimen volume and the measured porosity of 25%. For the consolidation test specimens, which were cylindrical with a base area of 30 cm^2^ and a height of 2 cm, the total specimen volume was 60 cm^3^, so the one-pore volume was 15 mL. For the slope model, which had a total sand volume of 2500 cm^3^, the one-pore volume was 625 mL. This injection volume allows the bacterial and cementation solutions to fully penetrate the sand under gravity. It also avoids surface runoff and loss of reactive materials caused by over-injection [40,41].

### 2.3. Specimen Preparation and MICP Treatment Procedure

For the consolidation tests, cylindrical molds with a base area of 30 cm^2^ and a height of 2 cm were used to pack the natural river sand. For the slope model, a straight channel model with a length of 25 cm and a width of 10 cm was constructed. The slope height was 10 cm, and the slope ratio was 1:2, with a total sand volume of 2500 cm^3^. All specimens were filled by natural pouring without additional compaction to maintain a consistent initial porosity of 25%. The MICP treatment was performed using a two-step injection method at room temperature (24 °C). First, the required volumes of bacterial suspension and cementation solution were measured according to each ratio listed in Table 1. The bacterial suspension was uniformly sprayed onto the sand surface or slope surface and allowed to stand for 15 min to promote microbial adsorption and colonization on sand particle surfaces [40,41]. Subsequently, the cementation solution was sprayed in the same manner. Both the cylindrical sand specimens for consolidation tests and the slope models for erosion tests were then left to stand for 24 h to allow the MICP reaction to proceed fully. During the treatment, all specimens were kept ventilated to maintain the aerobic conditions necessary for microbial metabolism. For the slope model, the total injection volume of 625 mL was proportionally divided into bacterial suspension and cementation solution according to the ratio listed in Table 1, and the treatment procedure was identical to that for the consolidation specimens. After the treatment, all specimens were cured at 24 °C for 24 h before subsequent testing.

### 2.4. Consolidation Test

The cured cylindrical sand specimens (base area 30 cm^2^, height 2 cm) were used for one-dimensional consolidation tests. A single-lever consolidation apparatus was used following standard geotechnical test procedures [42,43]. Loads were applied stepwise in the following order: 12.5, 25, 50, 100, 200, 300, and 400 kPa. After each load increment, the load was maintained until the deformation of the specimen stabilized (i.e., the dial gauge reading changed less than 0.005 mm per hour), and then the deformation was recorded. Based on the initial porosity (25%) and the initial specimen height (2 cm), the void ratio e corresponding to each load level was back-calculated from the measured deformation. The e-${log}_{10}p$ curve was then plotted with the logarithm of effective pressure (${log}_{10}p$) on the horizontal axis and the void ratio e on the vertical axis. The linear segment of the curve (50–400 kPa) was selected for linear regression, and the slope of the fitted line was taken as the compression index $C_{c}$. Each ratio was tested in triplicate, and the average value was reported.

### 2.5. Slope Erosion Test

To evaluate the effect of MICP treatment on the erosion resistance of the slope, scouring tests were conducted using a physical slope model. The slope model had a height of 10 cm, a length of 25 cm, and a slope ratio of 1:2 (vertical:horizontal). One side of the model was a glass wall for observation, and the other side was a sand slope formed by natural pouring. Two treatment groups were set up: a control group (treated with water only) and an experimental group (treated with the optimal ratio of 3:2). Each group was tested under two flow rates: 600 L/h and 1000 L/h. The scouring duration was 40 min, and the water level was kept constant at 4 cm. The scouring flow was supplied by a submersible pump, with water entering from the upstream end of the model and exiting from the downstream end. A stainless steel sieve with an aperture of 0.048 mm (300 mesh) was installed at the downstream outlet to collect the sand lost due to scouring. Every 10 min, the sand retained on the sieve was collected, air-dried for 24 h, and weighed to calculate the erosion mass for each time interval. The erosion modulus R was calculated by Equation (1):

(1) $$R=\frac{M}{At}$$

where M is the mass of sand lost during the scouring period (g), A is the eroded area of the slope (m^2^), and t is the scouring time (h). The unit of R is g/(m^2^·h).—The design of this erosion test refers to previous flume studies on MICP-treated sandy slopes [12,15,17,31,38].

## 3. Results

### 3.1. Effect of $r_{v}$ on $C_{c}$

**Figure 1:**
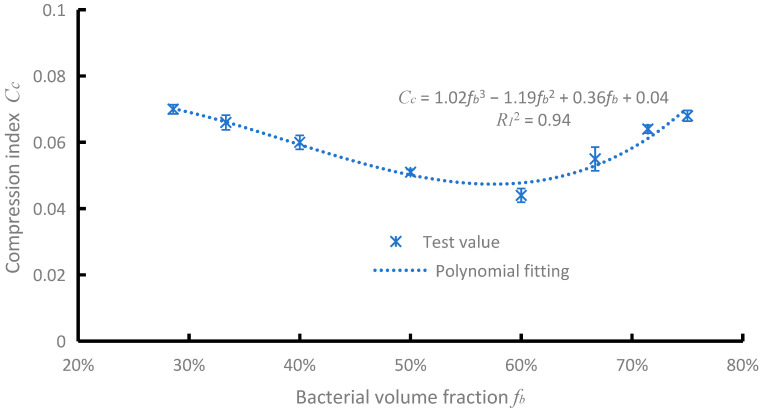
Variation in compression index ($C_{c}$) with bacterial volume fraction ($f_{b}$).

The compression index $C_{c}$ obtained from consolidation tests is summarized in Table 2. Figure 1 shows the variation of $C_{c}$ with bacterial volume fraction ($f_{b}$) for the eight tested ratios of bacterial solution to cementation solution. A clear non-linear “U-shaped” relationship is observed between $C_{c}$ and the bacterial volume fraction. The lowest $C_{c}$ value of 0.044 is achieved at $f_{b}$ = 60%, i.e., the ratio of 3:2 (bacterial solution:cementation solution). When $f_{b}$ > 70% or $f_{b}$ < 40%, $C_{c}$ increases significantly. Specifically, $C_{c}$ = 0.070 at 28.6% (2:5), 0.066 at 33.3% (1:2), 0.060 at 40.0% (2:3), 0.051 at 50.0% (1:1), 0.055 at 66.7% (2:1), 0.064 at 71.4% (5:2), and 0.068 at 75.0% (3:1). These results indicate that deviation from the optimal ratio leads to a marked deterioration in solidification performance and increased compressibility. The coefficient of determination *R*^2^ for all tests exceeded 0.98, confirming the high reliability of the calculated compression indices.

For reference, the untreated natural river sand (water-only treated) under identical consolidation conditions $C_{c}$ = 0.08 based on our preliminary tests.

One-way analysis of variance (ANOVA) was performed on the triplicate $C_{c}$ data. The results revealed a highly significant effect of $r_{v}$ on the compression index ($F_{7,16}$ = 58.6, p < 0.001). Post hoc Tukey HSD tests (α = 0.05) indicated that the optimal ratio (3:2, mean $C_{c}$ = 0.0443) yielded a significantly lower $C_{c}$ than all other ratios (all pairwise comparisons p < 0.05). The highest $C_{c}$ groups, 2:5 (mean 0.0700) and 3:1 (mean 0.0680), were not significantly different from each other (p > 0.05), but both were significantly higher than the 1:1 (0.0510), 2:1 (0.0550), and 2:3 (0.0600) groups (p < 0.05). These statistical results confirm the non-linear ‘U-shaped’ relationship and the distinct superiority of the 3:2 ratio.

To validate the non-linear ‘U-shaped’ relationship between $C_{c}$ and $f_{b}$, a polynomial fitting was conducted in a general form of:

(2) $$C_{c}=a{f_{b}}^{3}+b{f_{b}}^{2}+cf_{b}+d$$

For the specific conditions of this study (natural river sand, 0.063–1.6 mm, treated with Bacillus subtilis and cementation solution at 24 °C), the fitted coefficients are: a=1.02, b=−1.19, c=0.36, and d=0.04, with a coefficient of determination ${R_{1}}^{2}$ = 0.94. The minimum of this cubic curve occurs near $f_{b}$ = 60%, which coincides well with the experimentally determined optimal ratio $r_{v}$ = 3:2. It should be noted that this empirical model serves only to confirm the non-linear trend within the tested range and should not be extrapolated to other soil types, gradations, bacterial strains, or treatment conditions.

### 3.2. Microstructural Observation

**Figure 2:**
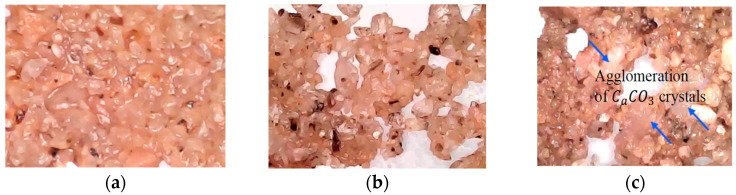
Microscopic images of sand particle surfaces after MICP treatment under different volume ratios: (**a**) $r_{v}$ = 3:2—continuous dense cementation layer; (**b**) $r_{v}$ = 3:1—sparse and isolated crystals; (**c**) $r_{v}$ = 2:5—local agglomeration and uneven precipitation.

To reveal the microscopic mechanism underlying the differences in compressibility among different ratios, the morphology of calcium carbonate on sand particle surfaces was observed using a digital microscope for three representative $r_{v}$ (3:2, 3:1, and 2:5). Figure 2 presents typical micrographs for these three ratios. At the optimal $r_{v}$ of 3:2 (Figure 2a), a continuous and dense calcium carbonate cementation layer formed on the sand particle surfaces, with crystals uniformly filling the inter-particle pores and showing no obvious defects. This uniform and dense cementation network effectively enhances inter-particle bonding, thereby reducing soil compressibility [19,44]. When the bacterial proportion was too high ($r_{v}$ = 3:1, $f_{b}$ = 75%) (Figure 2b), the calcium carbonate crystals were sparse and isolated, forming only a few localized cementation points, with many pores left unfilled. This is attributed to insufficient supply of cementation solution, leading to premature depletion of calcium ions and limited total calcium carbonate precipitation [45]. When the bacterial proportion was too low ($r_{v}$ = 2:5, $f_{b}=$ 28.6%) (Figure 2c), significant local agglomeration of calcium carbonate ($Ca{CO}_{3}$) was observed, with thick crystal clusters covering some sand particles while other areas remained nearly uncemented. This results from low urease activity due to insufficient bacterial supply, where the generation rate of carbonate ions cannot match the calcium ion concentration, leading to uneven precipitation [40]. These microstructural differences are consistent with the variation in compression index: the dense and continuous cementation network corresponds to the lowest $C_{c}$ (0.044), while sparse or agglomerated cementation structures lead to significantly higher $C_{c}$ values (0.068–0.070).

### 3.3. Erosion Resistance

**Figure 3:**
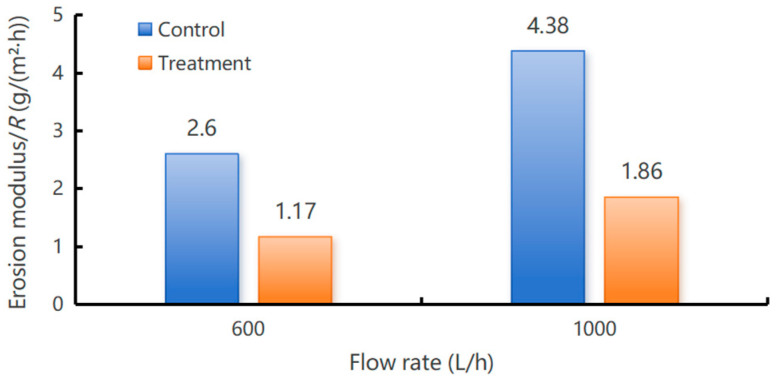
Bar chart comparing erosion modulus between control and MICP treatment groups under two flow rates (reduction of 55.0–57.5% for the treatment group).

To verify the engineering effectiveness of the optimal $r_{v}$ of 3:2, the erosion resistance of MICP-treated sand was evaluated using the slope scouring model. Figure 3 presents a comparison of the erosion modulus (R) between the control group (treated with water only) and the experimental group (MICP treated with the optimal ratio) under two flow rates (600 L/h and 1000 L/h). As shown in Table 3, at a flow rate Q = 600 L/h, R= 2.60 × 10^3^ g/(m^2^·h) for the control group, while for that of the experimental group it decreased to R= 1.17 × 10^3^ g/(m^2^·h), representing a reduction of 55.0%. At Q = 1000 L/h, R= 4.38 × 10^3^ g/(m^2^·h) for the control group, and for that of the experimental group R= 1.86 × 10^3^ g/(m^2^·h), representing a reduction of 57.5%. These results demonstrate that MICP treatment significantly enhances the erosion resistance of the slope.

**Figure 4:**
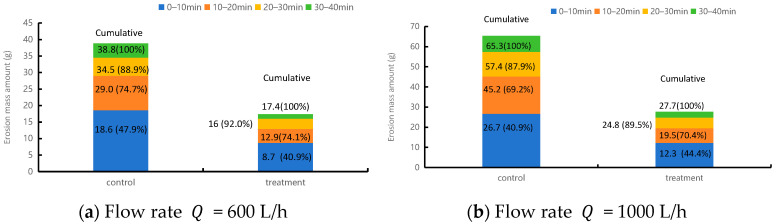
Temporal evolution of erosion mass for control and MICP treatment groups: (**a**) at flow rate of 600 L/h; (**b**) at flow rate of 1000 L/h.

Figure 4 shows the temporal variation in erosion mass during the scouring process. For the control group, the erosion mass was highest in the initial stage (0–10 min), accounting for 47.9% and 40.9% of the total erosion mass (*M*) at Q = 600 L/h and 1000 L/h, respectively, and then gradually decreased. In contrast, the erosion mass of the experimental group decayed more rapidly over time. Notably, in the later stage (30–40 min), the erosion mass of the experimental group accounted for only 8.0–10.5% of the total, which is much lower than the 11.1–12.1% observed for the control group. This indicates that the cementation network formed by MICP treatment not only protects the surface soil but also inhibits erosion of the deeper soil, accelerating the transition of the scouring system toward an equilibrium state.

### 3.4. Effect of $r_{v}$ on Slope Anti-Sliding Stability

**Figure 5:**
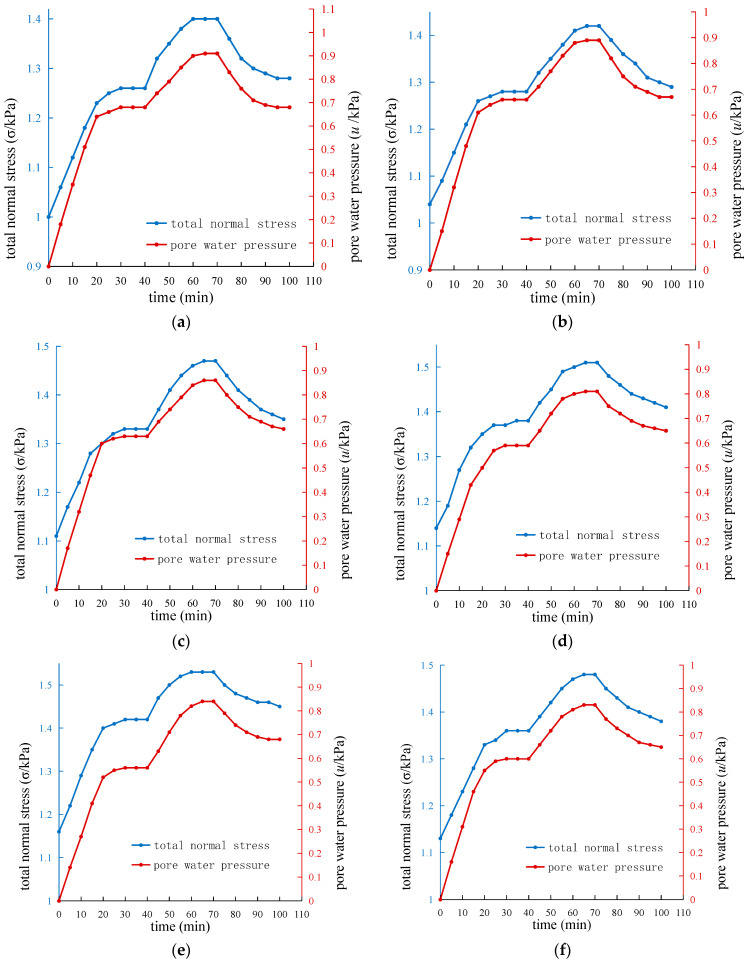

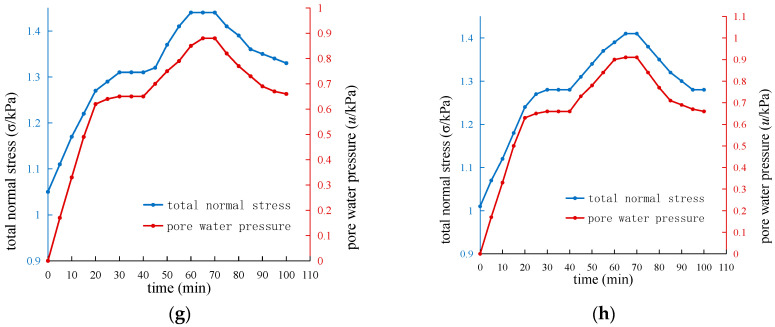
Temporal evolution curves of σ and u for the eight $r_{v}$: (**a**) $r_{v}$ = 2:5; (**b**) $r_{v}$ = 1:2; (**c**) $r_{v}$ = 2:3; (**d**) $r_{v}$ = 1:1; (**e**) $r_{v}$ = 3:2; (**f**) $r_{v}$ = 2:1; (**g**) $r_{v}$ = 5:2; and (**h**) $r_{v}$ = 3:1.

To evaluate the influence of different $r_{v}$ on the overall slope stability, the total normal stress σ and pore water pressure u were monitored in real time using embedded miniature soil pressure sensors and pore water pressure sensors under combined scouring-rainfall conditions. Figure 5a–h show typical evolution curves of σ and u over time for the eight $r_{v}$. All specimens exhibited a three-stage pattern: scouring stage (0–40 min), rainfall stage (40–70 min), and drainage stage (70–100 min). The steady-state scouring moment (40 min) and the peak rainfall moment (70 min) were selected as key analysis nodes.

**Figure 6:**
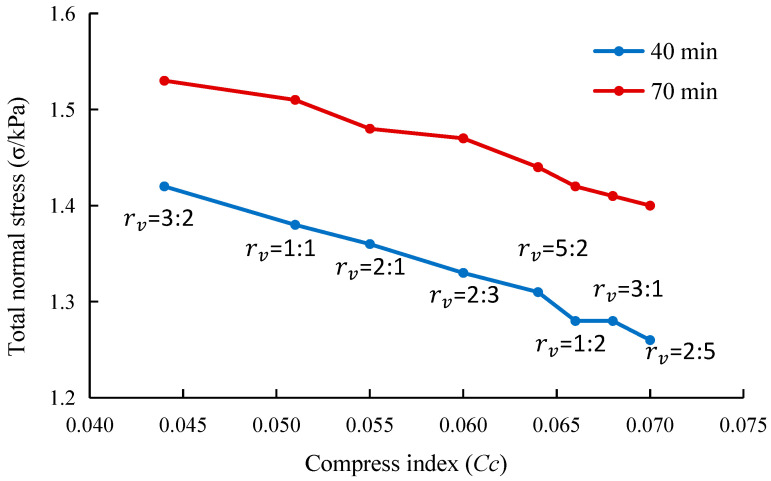
Relationship of σ–$C_{c}$.

Figure 6 presents the relationship between total normal stress σ and compression index $C_{c}$ at 40 min and 70 min. When $C_{c}$ decreased from 0.070 (2:5 ratio) to 0.044 (3:2 ratio), σ at 40 min increased from 1.26 kPa to 1.42 kPa, and *σ* at 70 min increased from 1.40 kPa to 1.53 kPa. The total normal stress showed a significant positive correlation with solidification efficiency, which can be attributed to the increased self-weight and improved structural integrity due to the cementation network.

**Figure 7:**
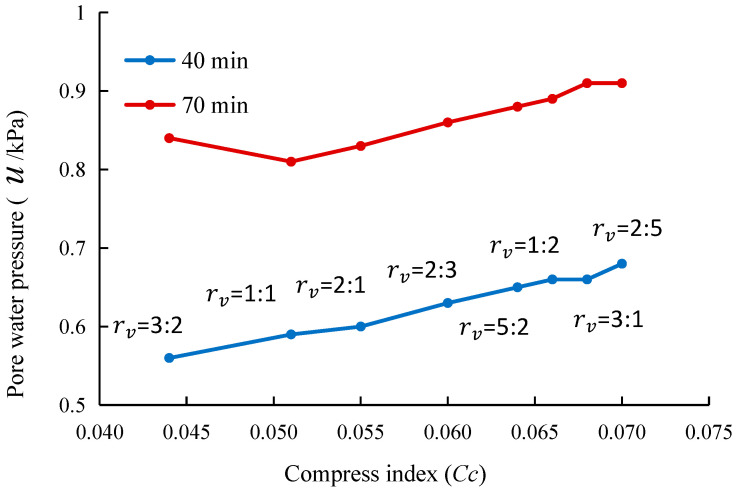
Relationship of $C_{c}$–u.

Figure 7 shows the relationship between pore water pressure u and $C_{c}$. During the scouring stage (40 min), u was positively correlated with $C_{c}$: the optimal ratio group exhibited the lowest u (0.56 kPa), while the high $C_{c}$ group (2:5) had the highest u (0.68 kPa). During the peak rainfall stage (70 min), except for the optimal ratio group, u remained positively correlated with $C_{c}$. However, the optimal ratio group showed a slightly higher u (0.84 kPa) than the 1:1 group (0.81 kPa), possibly due to surface ponding caused by an overly dense cementation network.

Based on the infinite slope model, the local anti-sliding stability coefficient K is given by:

(3) $$K=\frac{c+\left(\sigma -u\right)\tan\varphi }{\sigma \tan\beta }$$

where the cohesion c and internal friction angle φ were obtained by the consolidated quick direct shear test [18,46]; σ is the total normal stress; u is the pore-water pressure; β is the angle of slope.

Table 4 summarizes the shear strength parameters obtained from consolidated-quick direct shear tests. As $C_{c}$ decreased from 0.070 to 0.044, c increased from 2.1 to 4.8 kPa, and φ from 31.2° to 36.5°, indicating that MICP treatment enhanced both cohesion and frictional resistance.

**Figure 8:**
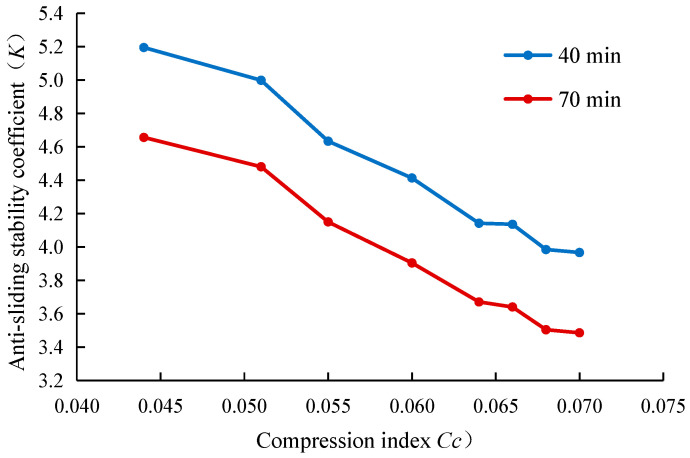
Relationship of K–$C_{c}$.

Figure 8 presents the K values for different ratios at the key nodes. When $C_{c}$ decreased from 0.070 to 0.044, K at 40 min increased from 3.97 to 5.19, and K at 70 min increased from 3.49 to 4.66. The anti-sliding stability coefficient showed a strong positive correlation with solidification efficiency, confirming that MICP treatment significantly enhances slope stability through dual pathways of increasing effective stress and shear strength parameters.

### 3.5. Ecological Performance: pH Variation

To assess the impact of MICP treatment on the soil chemical environment, the pH of the surface soil of the slope was measured at the end of the drainage stage (100 min) under combined scouring-rainfall conditions. The results showed a negative correlation between pH and solidification efficiency. The optimal ratio group (3:2) exhibited the lowest pH of 5.59, while the pH increased as solidification efficiency decreased, with the highest value of 6.32 observed for the 2:5 group. All pH values fell within the tolerance range (5.0–9.0) of typical slope vegetation such as vetiver grass. Rainfall and drainage processes caused a moderate increase in pH compared to the initial scouring stage, suggesting a regulating effect of hydraulic processes on the soil chemical environment.

## 4. Discussion

### 4.1. Mechanism of Ratio-Dependent Solidification Performance

The present study reveals a non-linear “U-shaped” relationship between the volumetric ratio of bacterial solution to cementation solution and the compression index of MICP-treated sand, with the optimal ratio identified as 3:2 (60% bacterial volume fraction). The underlying microscopic mechanism lies in the dynamic balance between urease activity and reactant supply [40,45]. At moderate bacterial volume fractions (50–66.7%), the urease provided by the bacteria hydrolyzes urea efficiently, and the generated carbonate ions match well with the calcium ions from the cementation solution. Calcium carbonate crystals nucleate and grow uniformly on sand particle surfaces and at contact points, eventually forming a continuous and dense cementation network [19]. This network effectively fills pore spaces and enhances inter-particle bonding, thereby reducing the compression index.

When the bacterial volume fraction is too high ($f_{b}$ > 70%), the cementation solution becomes relatively insufficient. Calcium ions and urea are consumed rapidly in the early stage, and the precipitation reaction ceases prematurely due to lack of reactants. Microscopic observation reveals sparse and isolated calcite crystals that fail to form a continuous network, leading to a higher compression index (3:1 group, $C_{c}$ = 0.068). When the bacterial volume fraction is too low ($f_{b}$ < 40%), urease activity is insufficient, and the generation rate of carbonate ions lags far behind the supply of calcium ions. This causes local supersaturation and rapid precipitation, forming agglomerated crystals (2:5 group, $C_{c}$ = 0.070), while most pore regions remain uncemented. These findings agree with DeJong et al. (2010) [44] and Cui et al. (2017) [19], who emphasized that the spatial distribution of calcium carbonate is more critical than its total amount in determining mechanical performance.

### 4.2. Enhancement of Erosion Resistance

The scouring tests show that the slope treated with the optimal ratio (3:2) exhibits a 55.0–57.5% reduction in erosion modulus compared to the control. This improvement can be attributed to three aspects. First, the dense calcium carbonate cementation layer strengthens the surface soil particles, making them less prone to detachment under hydraulic shear stress [12,31]. Second, the cementation network fills pores below the surface, reducing seepage pathways and inhibiting the development of internal erosion [15]. Third, because the surface strength is elevated, the initial erosion accounts for a larger proportion of the total erosion, while deeper soil experiences less disturbance, allowing the system to reach a scouring equilibrium more rapidly (Figure 4). This mechanism is consistent with the findings of Jiang et al. (2019) [17] on rainfall-induced erosion of MICP-treated sandy slopes.

### 4.3. Positive Correlation Between Anti-Sliding Stability and Solidification Efficiency

Based on infinite slope stability analysis, the factor of safety K increases significantly with improved solidification efficiency (Figure 7). The underlying reason is that MICP treatment enhances slope stability through two pathways. First, the calcite cement directly increases the cohesion c and internal friction angle φ of the soil, i.e., the shear strength parameters [46]. Second, the cementation network increases the self-weight of the soil (higher σ) and reduces pore water pressure *u* (especially during the scouring stage). According to the principle of effective stress $\sigma^{\prime }=\sigma -u$, the increased effective stress further contributes to shear strength. Notably, at the peak rainfall stage, the optimal ratio group showed a slightly higher pore water pressure (0.84 kPa) than the 1:1 group (0.81 kPa). This anomaly may be caused by the overly dense cementation layer hindering rapid rainwater infiltration, leading to temporary surface ponding. This suggests that in practical engineering, although high solidification efficiency is beneficial for stability, it may compromise drainage performance, and auxiliary drainage measures should be considered.

### 4.4. Engineering Implications and Limitations

This study provides a quantitative ratio guideline (bacterial solution:cementation solution = 3:2) for MICP solidification of natural river sand. This ratio significantly improves compressibility and erosion resistance while maintaining acceptable ecological compatibility (pH ranging from 5.59 to 6.32, within the tolerance range of most slope vegetation). Compared with previous studies using standard sand or artificially graded sand [18,47], the present work directly targets natural river sand, a common engineering material, making the conclusions more applicable to practice.

Nevertheless, several limitations should be acknowledged regarding the extrapolation of these laboratory-scale results to field conditions. First, the slope model is a reduced-scale physical model (10 cm in height); the geometry, stress levels, and boundary conditions of real-scale slopes may influence the long-term performance of MICP treatment. Second, the scouring tests were conducted under constant water level and unidirectional flow. The chosen flow rates (600 and 1000 L/h) correspond to average velocities of approximately 0.15 and 0.25 m/s in the flume (cross-section 0.1 m × 0.04 m), which are representative of moderate near-bank flows in medium-sized rivers (0.1–0.3 m/s) during normal discharge periods [2]. However, more complex hydrodynamic conditions—such as waves, water level fluctuations, extreme flood events, and long-term drying–wetting cycles—were not simulated, and may affect the durability of bio-cementation.

Third, the optimal ratio (3:2) was established specifically for the tested natural river sand with a defined gradation (0.063–1.6 mm) and porosity (25%). For sands with finer or coarser grains, the specific surface area and pore connectivity differ, which may shift the optimal ratio. For silty or clayey soils, the reduced permeability and higher surface charge could hinder bacterial transport and alter precipitation patterns, thus requiring site-specific re-optimization [21,24]. Therefore, the present ratio is not directly transferable to other soil types without further calibration.

Fourth, the ecological assessment was limited to preliminary pH measurements; long-term effects such as plant growth response, nutrient cycling, and microbial community evolution were not investigated. Future research should include full-scale field trials, multi-cycle scouring–rainfall–drying–wetting tests, and extended ecological monitoring to comprehensively evaluate the durability and environmental compatibility of MICP-solidified soils under realistic conditions.

## 5. Conclusions

This study systematically investigated the effect of the volumetric ratio of bacterial solution to cementation solution on the compressibility, erosion resistance, and slope stability of MICP-treated natural river sand. The main conclusions are as follows:

(1) The volumetric ratio exhibits a non-linear “U-shaped” influence on the compression index of solidified sand. The optimal ratio is 3:2 (bacterial solution:cementation solution), corresponding to a bacterial volume fraction of 60%, which yields the lowest compression index ($C_{c}$ = 0.044). Ratios with $f_{b}$ > 70% or $f_{b}$ < 40% lead to significantly higher $C_{c}$ values (≥0.064), indicating deteriorated solidification performance.
(2) Microstructural observations reveal that at the optimal ratio, a continuous and dense calcium carbonate cementation network forms on sand particle surfaces, uniformly filling the pore spaces. Excess bacterial solution results in sparse cementation, whereas insufficient bacterial solution leads to local agglomeration. The spatial distribution of calcium carbonate is a key factor determining solidification efficiency.
(3) The slope treated with the optimal ratio (3:2) exhibits a 55.0–57.5% reduction in erosion modulus (R) compared to the untreated control. The erosion mass decays more rapidly over time, and the scouring system reaches equilibrium faster. MICP enhances erosion resistance through dual mechanisms: strengthening the surface layer against detachment and inhibiting internal seepage erosion.
(4) The factor of safety against sliding (K) shows a strong positive correlation with solidification efficiency. As $C_{c}$ decreases from 0.070 to 0.044, K at the steady scouring stage (40 min) increases from 3.97 to 5.19, and K at the peak rainfall stage (70 min) increases from 3.49 to 4.66. MICP enhances slope stability through two pathways: increasing effective stress ($\sigma^{\prime }=\sigma -u$) and improving shear strength parameters (c, φ). However, excessive solidification may reduce permeability and impair drainage efficiency.
(5) The $r_{v}$ value of 3:2 is recommended for MICP solidification of natural river sand in engineering practice. This ratio significantly improves compressibility and erosion resistance, with measured soil pH ranging from 5.59 to 6.32, which falls within the tolerance range of most slope vegetation. In practical applications, auxiliary drainage measures are advisable to balance mechanical performance and ecological adaptability.

## Figures and Tables

**Table 1:** Volumetric ratios of bacterial solution to cementation solution.

Bacterial: Cementation $r_{v}$	Bacterial Volume Fraction $f_{b}$ (%)	Injection Volume for Consolidation Test (mL)	Injection Volume for Slope Model (mL)
2:5	28.6	15	625
1:2	33.3		
2:3	40.0		
1:1	50.0		
3:2	60.0		
2:1	66.7		
5:2	71.4		
3:1	75.0		

Note: The volumes of bacterial solution and cementation solution were proportionally allocated according to $r_{v}$, with the total injection volume fixed at one pore volume of the corresponding specimen.

**Table 2:** Compression index of MICP-treated sand under different volume ratios.

Bacterial:Cementation $r_{v}$	Bacterial Volume Fraction $f_{b}$ (%)	Compression Index $C_{c}$	SD	95% CI
2:5	28.6	0.070	0.0014	[0.06656, 0.07344]
1:2	33.3	0.066	0.0022	[0.06054, 0.07146]
2:3	40.0	0.060	0.0021	[0.05479, 0.06521]
1:1	50.0	0.051	0.0009	[0.04877, 0.05323]
3:2	60.0	0.044	0.0021	[0.03917, 0.04949]
2:1	66.7	0.055	0.0036	[0.04604, 0.06396]
5:2	71.4	0.064	0.0012	[0.06099, 0.06701]
3:1	75.0	0.068	0.0016	[0.06413, 0.07187]

**Table 3:** Erosion modulus of slopes under different treatments.

Treatment	Flow Rate Q (L/h)	Total Erosion Mass M (g)	Erosion Modulus R (g/(m^2^·h))
Control	600	38.8	2.60 × 10^3^
Control	1000	65.3	4.38 × 10^3^
Experimental	600	17.4	1.17 × 10^3^
Experimental	1000	27.7	1.86 × 10^3^

**Table 4:** Porosity and shear strength indices (*c*, *φ*) of bio-cemented sand under different mixing ratios.

$r_{v}$	Porosity (%)	*c* (kPa)	*φ* (°)
2:5	0.243	2.12	33.17
1:2	0.238	2.22	34.55
2:3	0.231	2.43	35.80
1:1	0.222	2.87	36.23
3:2	0.216	3.05	36.58
2:1	0.228	2.60	35.93
5:2	0.236	2.25	35.06
3:1	0.242	2.14	33.48

## Data Availability

The original contributions presented in this study are included in the article. For further inquiries, please contact the corresponding authors.
